# Supplementary material for: Biodiversity-Friendly Management in Olive Groves Supports Pollinator Conservation in a Mediterranean Terraced Landscape
Source: Insects. 2025 Feb 12;16(2):198. doi: 10.3390/insects16020198 (PMC11856615; doi:10.3390/insects16020198)
Supplement: Supplementary file 1 [file insects-16-00198-s001.zip › Supplementary.pdf]

Supplementary materials

Table S1: overview of the sampling of solitary bees, bumblebees, flowers and butterflies across both years and all sampling times and habitats.

	2021												2022											
	T1			T2			T3			T4			T1			T2			T3			T4		
	OL	HL	WA				OL	HL	WA	OL	HL	WA	OL	HL	WA	OL	HL	WA	OL	HL	WA	OL	HL	WA
Solitary bees	30	33	10	NA	NA	NA	15	13	20	NA	NA	NA	31	28	12	38	81	16	39	32	28	7	8	6
Bumblebees	1	1	3	NA	NA	NA	1	1	0	NA	NA	NA	0	0	0	4	0	1	3	4	2	0	1	2
Flowers	4638	3515	1381	NA	NA	NA	419	479	253	NA	NA	NA	11728	4956	2328	1760	313	649	703	882	227	322	287	138
Butterflies	NA	NA	NA	120	89	69	26	25	20	36	45	75	69	50	22	108	80	74	35	24	17	9	19	11

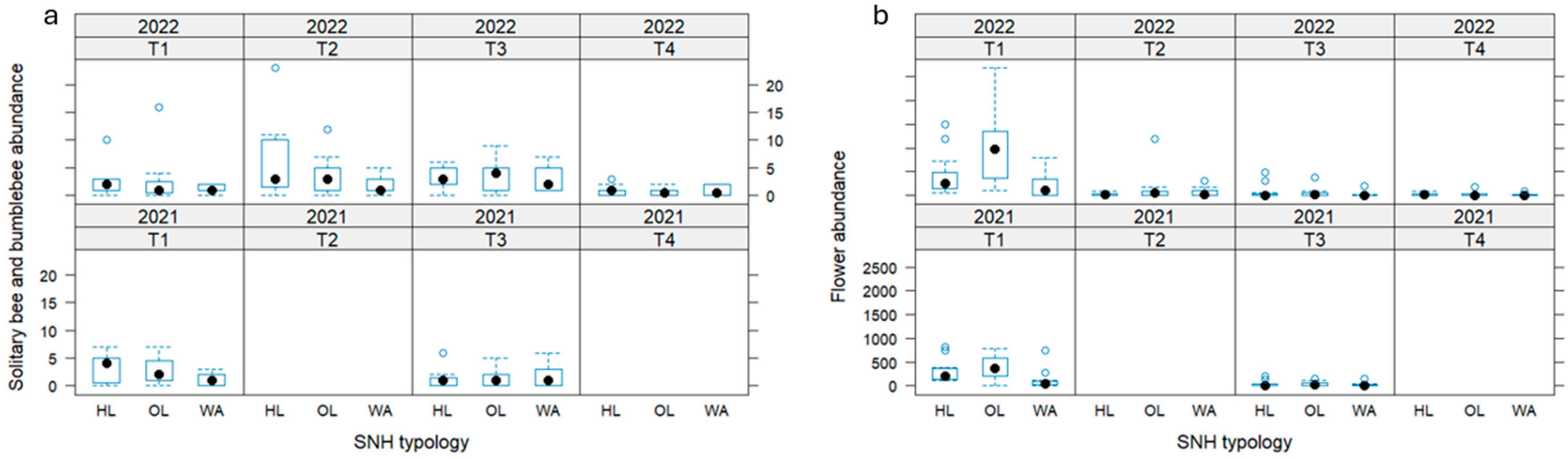

Figure S1: boxplots representing solitary bee and bumblebee abundances (a) and flower abundances (b) across both years and all sampling times and habitats.

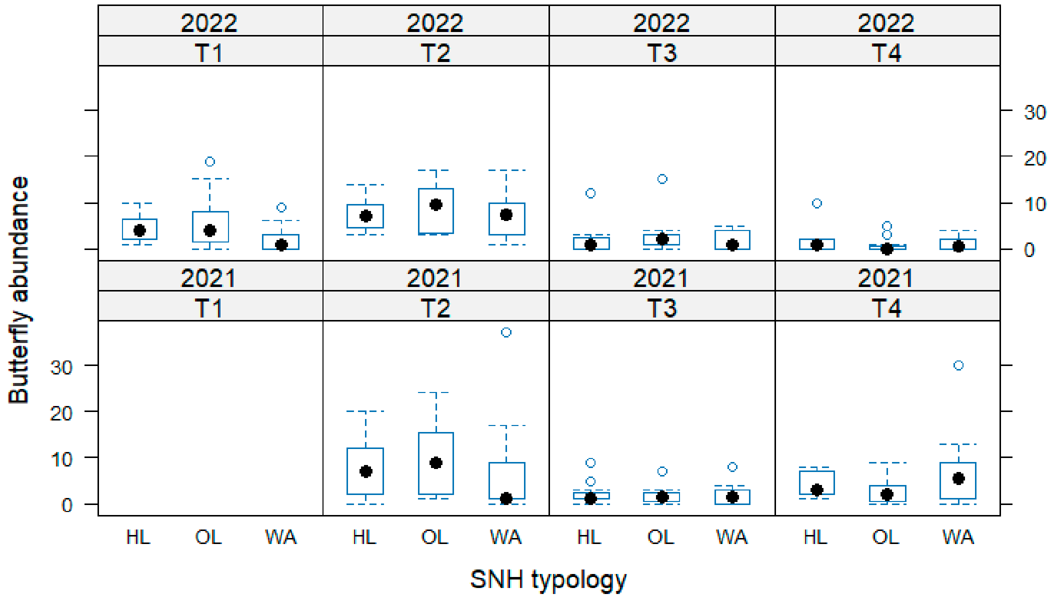

Figure S2: boxplots representing butterfly abundance across both years and all sampling times and habitats.

Table S2: overview of the flower sampling with all species and abundances recorded in the two years across all sampling times and habitats

	2021									2022											
	T2			T3			T4			T1			T2			T3			T4		
	HL	OL	WA	HL	OL	WA	HL	OL	WA	HL	OL	WA	HL	OL	WA	HL	OL	WA	HL	OL	WA
Anthocharis cardamines	0	1	0	0	0	0	0	0	0	0	0	1	0	0	0	0	0	0	0	0	0
Gonepteryx rhamni	1	0	0	0	0	0	0	0	0	0	0	0	0	1	0	0	0	0	0	0	0
Limenitis reducta	0	0	1	0	0	0	2	0	4	0	0	0	0	0	1	0	0	2	1	0	1
Coenonympha arcania	0	4	2	0	0	0	1	5	0	0	0	0	0	0	1	0	0	0	0	0	0

Colias crocea	13	9	6	1	1	0	1	0	1	2	1	0	9	6	4	0	0	1	0	0	0
Vanessa cardui	1	1	2	0	0	0	1	0	0	4	11	3	0	0	0	0	0	0	0	0	0
Lasiommata megera	0	11	0	2	7	6	6	3	4	1	1	2	6	7	10	1	2	1	2	1	1
Aricia agestis	0	1	0	1	1	2	5	5	6	1	4	0	2	5	1	0	0	1	0	0	0
Pontia edusa	0	0	4	3	4	1	0	0	0	0	0	0	0	0	0	0	0	0	0	0	0
Papilio machaon	1	4	13	2	0	1	2	0	3	1	0	0	4	2	6	2	2	1	2	0	0
Euchloe ausonia	1	0	1	0	0	0	0	0	0	0	0	0	0	0	0	0	0	0	0	0	0
Polygonia c.album	0	0	0	0	0	0	0	0	1	0	0	0	0	0	0	0	0	0	0	0	0
Iphiclides podalirius	0	0	0	1	0	0	0	0	0	1	0	0	1	2	3	0	0	0	0	0	0
Pieris mannii	4	2	0	0	0	0	0	0	0	0	0	0	1	0	0	0	1	0	0	0	0
Thymelicus sylvestris	0	2	0	0	0	0	0	0	0	1	7	1	2	7	0	1	0	0	1	0	0
Ochlodes sylvanus	0	0	0	0	0	0	1	2	1	0	0	0	0	0	0	0	0	0	0	0	0
Leptotes pirithous	0	0	0	0	0	0	1	1	1	0	0	0	0	0	0	0	0	0	4	0	0
Satyrium ilicis	0	1	1	0	0	0	0	0	0	0	0	0	0	0	0	0	0	0	0	0	0
Pyrgus malvoides	0	0	0	0	0	0	0	0	0	2	0	0	1	3	0	1	0	0	0	0	0
Limenitis camilla	0	0	0	0	0	0	0	0	0	0	0	0	1	1	0	0	0	3	0	0	0
Coenonympha pamphilus	0	1	0	0	0	0	0	0	0	9	6	0	3	3	2	1	4	0	1	0	1
Vanessa atalanta	1	1	0	0	0	0	0	0	1	0	1	2	0	0	0	0	0	0	0	0	0
Pararge aegeria	0	0	1	1	0	0	1	0	5	2	1	2	0	0	6	0	0	0	0	0	0
Favonius quercus	0	0	0	0	0	0	0	0	1	0	0	0	0	0	0	0	0	0	0	0	0
Polyommatus icarus	2	4	1	4	4	1	5	5	22	8	15	0	7	15	2	6	13	4	1	0	0
Melitaea didyma	0	0	2	6	0	0	0	0	0	0	0	0	6	7	5	1	0	0	0	0	0
Argynnis paphia	0	0	0	0	0	0	1	0	3	0	0	0	0	0	0	0	0	0	0	0	0
Melitaea phoebe	0	0	1	0	0	0	0	0	0	0	0	0	0	0	0	0	0	0	0	0	0
Maniola jurtina	19	32	5	0	0	1	4	8	4	3	1	0	14	24	10	1	0	2	3	0	3
Pieris rapae	7	4	0	0	5	0	0	0	0	7	5	3	4	2	0	5	2	0	0	0	0

Lycaena tityrus	1	1	0	0	0	0	0	0	0	0	0	0	0	0	0	0	0	0	0	0	0
Hipparchia statilinus	0	0	0	0	0	0	10	3	10	0	0	0	0	0	0	1	2	0	3	6	4
Pieris napi	1	1	0	0	0	0	2	0	1	0	0	0	0	1	0	1	0	0	0	0	0
Melanargia galathea	7	23	1	0	0	5	0	0	0	0	0	0	16	10	11	0	0	0	0	0	0
Pyronia cecilia	0	0	0	0	2	1	0	0	0	0	0	0	0	0	0	1	5	0	0	0	0
Gonepteryx cleopatra	2	2	10	2	0	0	2	0	1	0	0	0	0	3	5	0	2	1	0	0	1
Muschampia sp.	0	0	0	0	1	0	0	0	0	0	0	0	0	0	0	0	0	0	0	0	0
Hipparchia fagi	0	0	0	0	0	0	0	1	2	0	0	0	1	0	0	0	0	0	0	1	0
Satyrium acaciae	0	0	1	0	0	0	0	0	0	0	0	0	0	0	0	0	0	0	0	0	0
Pieris brassicae	23	13	13	0	1	1	0	0	0	6	12	7	0	1	2	1	1	1	0	0	0
Lampides boeticus	0	0	0	0	0	0	0	1	1	0	0	0	0	0	0	0	0	0	0	0	0
Cacyreus marshalli	0	0	0	0	0	0	0	1	0	0	0	0	0	0	0	0	0	0	0	0	0
Callophrys rubi	0	0	1	0	0	0	0	0	0	0	1	0	0	0	1	0	0	0	0	0	0
Pontia daplidice	2	0	0	0	0	0	0	0	0	0	0	0	0	0	0	0	0	0	0	0	0
Leptidea sinapis	0	0	1	0	0	0	0	0	0	0	0	0	0	0	0	0	0	0	0	0	0
Charaxes jasus	0	0	0	0	0	0	0	0	1	0	0	0	0	0	0	0	0	0	0	0	0
Lycaena phlaeas	0	1	1	0	0	1	0	0	0	2	2	0	2	7	2	1	0	0	1	1	0
Celastrina argiolus	0	0	1	1	0	0	0	1	1	0	1	1	0	0	2	0	0	0	0	0	0
Macroglossum stellatarum	3	1	0	1	0	0	0	0	1	0	0	0	0	1	0	0	1	0	0	0	0

Table S3: overview of the butterfly sampling with all species and abundances recorded in the two years across all sampling times and habitats

	2021						2022											
	T1			T3			T1			T2			T3			T4		
	HL	OL	WA	HL	OL	WA	HL	OL	WA	HL	OL	WA	HL	OL	WA	HL	OL	WA
Achillea millefolium	37	60	0	0	0	0	72	50	0	0	6	0	0	0	0	0	0	0
Allium sativum	0	0	0	0	0	0	0	0	0	0	0	0	0	7	0	0	0	0
Anagallis arvensis	73	61	8	0	0	0	45	93	0	0	0	0	0	0	0	0	0	0
Anthemis arvensis	156	0	0	0	0	0	85	0	0	0	0	0	0	0	0	0	0	0
Anthyllis vulneraria	0	0	2	0	0	0	0	0	0	0	0	0	0	0	0	0	0	0
Artemisia vulgaris	0	0	0	0	0	0	121	0	0	0	0	0	0	0	0	0	0	0
Asteracee yellow	1,253	1,879	652	139	249	51	1,981	4,938	1,305	175	1,152	140	256	429	127	69	189	11
Ballota nigra	116	184	87	0	6	32	0	0	0	0	40	0	0	30	0	0	0	0
Bellardia viscosa	0	0	0	0	0	0	0	10	0	0	0	0	0	0	0	0	0	0
Bellis perennis	4	0	15	2	2	0	32	0	0	0	0	0	0	0	0	0	0	0
Beta vulgaris	0	0	0	0	0	0	8	0	0	0	0	0	0	0	0	0	0	0
Blackstonia perforata	0	2	0	0	0	0	0	0	0	0	6	0	0	0	0	0	0	0
Calamintha nepeta	3	0	0	198	115	0	0	0	0	0	0	0	12	18	0	61	35	0
Campanula rapunculus	323	72	47	0	0	0	2	7	3	20	18	0	0	0	2	0	0	0
Carduus pycnocephalus	133	0	110	0	0	0	7	21	17	2	0	0	0	0	0	0	0	0
Castanea sativa	0	0	0	0	0	0	0	0	0	0	0	30	0	0	0	0	0	0
Centaurea jacea	0	1	0	2	0	0	0	0	3	0	0	10	0	0	0	0	0	0
Centaurium erythraea	11	44	0	0	0	0	0	0	0	8	60	0	0	0	0	0	0	0
Centaurium maritimum	0	2	0	0	0	0	0	22	0	0	0	0	0	0	0	0	0	0
Centranthus ruber	0	0	0	0	0	0	0	0	83	0	0	0	0	0	0	0	0	0

Cichorium endivia	0	0	0	0	0	0	0	0	0	0	0	0	0	0	0	0	0	0
Cichorium intybus	0	2	0	2	4	0	0	0	0	0	0	0	10	0	11	0	0	0
Cirsium vulgare	99	56	0	0	0	0	331	30	0	0	0	0	0	0	0	0	0	0
Cistus salvifolius	0	0	0	0	0	0	0	0	2	0	0	0	0	0	0	0	0	0
Convolvulus arvensis	29	49	0	0	0	0	21	1	0	10	58	0	11	19	0	0	0	0
Cyclamen hederifolium	0	0	0	0	0	150	0	0	0	0	0	0	0	0	0	0	0	95
Cymbalaria muralis	0	0	0	0	0	0	21	0	0	0	0	0	0	0	0	0	0	0
Daphne gnidium	0	0	0	0	0	0	0	0	0	0	0	50	0	0	0	0	0	0
Daucus carota	154	294	11	39	4	0	0	11	0	0	18	0	15	8	0	55	10	2
Dianthus carthusianorum	1	2	0	3	0	2	0	0	0	0	46	6	26	69	0	7	36	1
Dorycnium hirsutum	0	0	12	0	0	0	0	0	235	0	0	0	0	0	0	0	0	0
Echium italicum	0	1	0	0	0	0	0	0	0	8	0	0	30	0	0	0	0	0
Echium vulgare	12	0	0	0	0	0	0	1	0	0	0	0	0	0	0	0	0	0
Erigeron annuus	0	0	2	0	0	0	0	0	0	0	0	0	0	0	0	0	0	0
Fumaria officinalis	8	0	0	0	0	0	20	113	0	0	0	0	0	0	0	0	0	0
Galeopsis tetrahit	0	0	0	0	0	0	0	0	0	0	0	0	0	0	0	0	0	8
Galium aparine	0	146	245	0	0	0	0	150	0	0	0	0	0	0	0	0	0	0
Galium mollugo	30	311	0	0	0	0	60	58	0	0	0	0	0	0	0	0	0	0
Genista pilosa	0	0	0	0	0	0	0	0	0	0	0	0	0	0	0	0	0	1
Geranium dissectum	19	22	6	0	0	0	0	115	3	0	0	0	0	0	0	0	0	0
Geranium molle	70	1	11	0	0	0	304	254	9	1	0	0	0	0	0	0	0	0
Geum urbanum	0	0	0	0	0	0	0	2	1	0	0	0	0	0	0	0	0	0
Hedysarum coronarium	0	0	0	0	0	0	0	0	1	0	0	0	0	0	0	0	0	0
Hypericum perforatum	3	18	9	0	0	2	0	2	0	0	115	0	3	60	0	0	0	0
Kickxia spuria	20	0	0	0	0	0	0	0	0	0	0	0	0	0	0	0	0	0
Knautia arvensis	0	5	0	0	0	0	0	0	0	0	0	0	0	0	0	0	0	0

Ligustrum vulgare	0	0	0	0	0	0	0	0	0	0	0	318	0	0	0	0	0	0
Linum strictum	0	0	0	0	0	0	0	0	23	0	0	0	0	0	0	0	0	0
Linum trigynum	0	2	0	0	0	0	0	60	0	0	0	0	0	2	0	0	0	0
Linum usitatissum	0	0	2	0	0	0	4	65	0	0	0	0	0	0	0	0	0	0
Lonicera caprifolium	0	0	7	0	0	0	0	0	17	0	0	0	0	0	0	0	0	0
Lotus corniculatus	25	62	0	3	0	0	0	89	26	0	0	0	0	0	0	0	0	0
Lychnis flos.cuculi	0	0	0	0	0	0	0	33	0	0	0	0	0	0	0	0	0	0
Malva pratensis	0	1	0	0	0	0	0	0	0	0	41	0	0	0	0	3	0	0
Matricaria chamomilla	0	4	0	0	0	0	0	0	0	0	0	0	0	0	0	0	0	0
Matricaria officinalis	0	0	0	0	0	0	0	225	45	0	0	0	14	0	0	0	0	0
Medicago minima	5	96	0	0	0	0	13	8	0	0	0	0	0	0	0	0	0	0
Melilotus indicus	0	0	0	0	0	0	0	0	145	0	0	0	0	0	0	0	0	0
Mentha spicata	0	0	0	0	0	0	0	0	0	0	0	0	0	0	0	24	0	0
Micromeria graeca	0	0	0	0	0	0	12	0	0	0	0	0	0	0	0	0	0	0
Mirtus communis	0	0	0	0	0	0	0	0	0	0	0	35	0	0	0	0	0	0
Myosotis arvensis	14	0	0	0	2	0	7	15	0	0	0	0	0	0	0	0	0	0
Nerium oleander	0	0	0	0	0	0	0	0	0	60	0	0	480	0	0	15	0	0
Oenanthe crocata	0	0	0	0	0	0	7	72	0	0	17	0	0	0	0	0	0	0
Ornithopus compressus	0	0	0	0	0	0	0	52	0	0	0	0	0	0	0	0	0	0
Orobanche hederae	2	4	2	0	0	0	23	7	0	0	0	0	0	0	0	0	0	0
Oxalis acetosella	0	28	0	0	4	0	9	70	0	0	0	0	0	0	0	0	0	0
Oxalis pescaprae	0	0	0	0	5	0	0	3	0	0	0	0	0	0	0	0	0	0
Papaver rhoeas	4	18	0	0	0	0	7	0	4	0	0	0	0	0	0	0	0	0
Parietaria officinalis	0	0	0	0	0	0	0	9	0	0	0	0	0	0	0	0	0	0
Passiflora caerulea	0	0	0	0	0	0	0	8	0	0	0	0	0	0	0	0	0	0
Petrorhagia saxifraga	0	0	0	0	0	0	0	0	0	0	0	0	6	0	0	0	0	0
Phagnalon sordidum	0	0	0	0	0	0	110	0	0	0	0	0	0	0	0	0	0	0

Phoeniculum vulgare	0	0	0	14	0	0	0	0	0	0	0	0	0	25	0	18	1	0
Plantago lanceolata	95	29	0	0	0	0	0	0	0	7	0	0	0	0	0	0	0	0
Polygonum aviculare	0	0	0	0	0	0	0	0	0	0	4	0	0	0	0	0	0	0
Potentilla reptans	40	10	3	0	0	0	51	20	0	0	0	0	0	0	0	0	0	0
Prunella laciniata	21	17	0	0	0	0	0	0	0	0	0	0	0	0	0	0	0	0
Ranunculus bulbosus	0	0	6	0	0	0	0	1	0	0	0	0	0	0	0	0	0	0
Ranunculus repens	0	0	0	0	0	0	0	29	0	0	0	0	0	0	0	0	0	0
Raphanus raphanistrum	138	19	26	0	0	0	170	231	0	0	2	0	0	0	0	0	0	0
Rosa canina	0	0	0	0	0	0	0	0	110	0	0	0	0	0	0	0	0	0
Rubia peregrina	0	0	0	0	0	0	0	0	22	0	0	0	0	0	0	0	0	0
Rubus ulmifolius	4	12	22	0	0	0	1	0	0	10	2	60	0	0	11	0	0	0
Rumex crispus	2	0	0	0	0	0	36	14	0	0	0	0	0	0	0	0	0	0
Sanguisorba minor	0	0	0	0	0	0	96	22	0	0	0	0	0	0	0	0	0	0
Scabiosa triandra	0	0	0	0	0	0	0	0	0	6	0	0	0	4	0	0	0	0
Serapias lingua	0	4	0	0	10	0	0	4	0	0	0	0	0	0	0	0	0	0
Sherardia arvensis	0	41	0	0	0	0	61	187	0	0	0	0	0	0	0	0	0	0
Silene vulgaris	3	57	19	2	0	0	33	74	94	1	10	0	3	5	0	1	2	0
Sinapis arvensis	0	43	0	0	0	0	95	236	20	0	0	0	0	0	0	0	0	0
Solanum nigrum	3	8	0	15	57	0	0	2	0	0	0	0	0	19	0	0	13	0
Stellaria media	0	3	0	0	0	0	0	22	0	0	0	0	0	0	0	0	0	0
Sysimbrium officinalis	0	0	0	0	0	0	0	25	0	0	0	0	0	0	0	0	0	0
Tamarix aphilla	0	0	0	0	0	0	0	0	0	0	0	0	0	0	76	0	0	0
Torilis arvensis	0	0	0	0	0	0	0	11	0	0	0	0	0	0	0	0	0	0
Tradescantia fluminensis	0	0	52	0	0	16	0	435	0	0	0	0	0	0	0	0	0	0
Trifolium arvense	212	101	0	0	0	0	188	60	0	0	150	0	0	0	0	0	0	0
Trifolium campestre	50	64	9	0	0	0	287	1,615	60	0	0	0	0	0	0	0	0	0
Trifolium glomeratum	3	0	3	0	0	0	0	60	0	0	0	0	0	0	0	0	0	0

Trifolium pratense	3	4	5	0	0	0	0	0	25	5	6	0	0	0	0	0	0	0
Trifolium repens	336	329	0	0	0	0	557	404	0	0	0	0	0	0	0	0	0	0
Trifolium subterraneum	0	30	0	0	0	0	44	233	21	0	0	0	0	0	0	0	0	0
Umbilicus rupestris	0	0	0	0	0	0	9	0	0	0	0	0	0	0	0	0	0	0
Urtica dioica	0	7	8	0	0	0	0	4	0	0	0	0	0	0	0	0	0	0
Verbascum nigrum	0	0	0	0	0	0	0	0	0	0	0	0	0	0	0	14	0	20
Verbena officinalis	1	1	0	0	21	0	0	0	8	0	1	0	16	0	0	20	36	0
Veronica persica	0	0	0	0	0	0	0	8	0	0	0	0	0	0	0	0	0	0
Vicia cracca	0	428	0	0	0	0	0	0	0	0	0	0	0	0	0	0	0	0
Vicia lutea	0	4	0	0	0	0	26	1,437	46	0	8	0	0	8	0	0	0	0
